# Spirit of the Foreign Periodicals, &c.

**Published:** 1845-01

**Authors:** 


### Periscope;

OR,

### CIRCUMSPECTIVE REVIEW.

" Ore trahit quodcunque potest, atque addit acervo."

### Spirit of the Foreign Periodicals.

### A New Classification of Cutaneous Diseases.

M. Devergie is one of the Physicians of the St. Louis Hospital in Paris, and enjoys, therefore, an immense experience in the observation and treatment of cutaneous diseases. As a reason for proposing the following arrangement, he says that " every day medical practitioners send to me patients affected with what they most vaguely call *dartres,* without the species of the disease being even so much as named. This much-abused appellation unfortunately always suggests the use of sulphureous and depurative medicines—a class of remedies which very often exasperates, instead of curing, the existing skin disease."

*Nosological Table of Cutaneous Diseases.*

I. Secreting Diseases.

A. *Serosity.*
Eczema.
Pityriasis rubra acuta.
Eczema lichenoides.
Scabies (gale).
Herpes phlyctenoides.
Pemphigus.

B. *Purulent serosity.*
Eczema impetiginodes.

C. *Purulent and sanious serosity.*
Rupia.
Ecthyma cachecticum.

D. *Pus.*
Impetigo.
Acne.
Scabies (gale).
Ecthyma.
Sycosis.

E. *Fatty matter.*
Acne sebacea.
Acne punctata.

II. Non-Secreting Diseases.

A. *Transitory* redness.*
Erythema.
Urticaria.
Roseola.
" Couperose erythemateuse."

B. *Permanent redness.*
Purpura.
Scorbutus.

C. *Redness with a papular state of the skin.*
Lichen.
Strophulus.

D. *Circumscribed redness with furfuraceous scales and a fretted state of the skin.*
Herpes circinatus.
Herpes nummularis.

E. *Diffused redness with a furfuraceous state of the skin.*
Pityriasis rubra.

F. *Redness with induration of the skin and with scales.*
Psoriasis.
Lepra vulgaris.

* *i. e.* Disappearing under the pressure of the finger.

G. *Scales without redness.*
Icthyosis.

H. *Papulæ without redness.*
Lichen chronica.
Prurigo.

I. *Vegetable productious.*

K. *Animal productions.*

A. In *eczema,* the serous secretion oozes from a red surface, by myriads of little canals or openings, in the form of minute drops, or fine moisture.

In *acute pityriasis* the serous oozing is a sort of sweat, but without any perceptible punctuated state of the skin.

In *eczema lichenoides,* the serosity proceeds from a rounded patch, which is sprinkled over with a multitude of papulæ that render the surface somewhat rough. The discharge is exhaled between the papulæ.

In *scabies,* the serosity is contained in minute isolated Vesicles; whereas,

In *herpes phlyctenoides,* it is within Bullæ, that are either distinct and separate, or are situated so near to each other as almost to touch. In *pemphigus,* the bullæ are still larger.

B. There is one disease only, viz. *eczema impetiginodes,* in which the secretion is partly serous and partly purulent at the same time. The product of this secretion is a crust, of a yellowish-grey colour.

C. In *rupia* and *ecthyma cachecticum* the secreted matter is partly purulent and partly sanious. In the former, the crust, that is subsequently formed, has an ill-favoured appearance—being formed of pus, ichor, and blood—and is always more or less irregular in its form. In the latter, the crust exhibits the same characters, but it rests upon an inflamed base, and retains the rounded shape of the pustule which preceded it.

D. Among the cutaneous diseases in which the secretion consists entirely of pus, *impetigo* is alike the most common and most important: its crusts are always purulent, superficial, and of a gold-yellow colour, not unlike that of concrete honey.

In *acne,* the pustules are hard at the base, and elevated; in *purulent scabies,* they are flat and more uniform. In *ecthyma,* the pustules are flat and of a large size, and exhibit in the middle a dark point, which gradually becomes more and more depressed, so that at length they are umbilicated, as in small-pox.

We must pass over the remaining family of this section, and all those of the other or non-secreting class, till we come to the last two groupes of the series—viz. the Vegetable and Animal Productions of the skin. Among the former, are enumerated the three varieties of genuine *tinea* or *favus,* viz. *f. scutulata, lupinosa,* and *granulata;* also the *porrigo decalvans,* and *herpes tonsurans.* Much skill and experience are required to discriminate these affections.

The *animal productions* comprise the *pedicular* disease, and that which engenders the *pulex* or flea.

M. Devergie promises to shew, in a subsequent article, the practical bearings, in a therapeutic as well as in a diagnostic point of view, of the classification which he has proposed:—the treatment required for *secreting* diseases of the skin being very different from that which is suited to those that are *non-secreting.*—*Bulletin General de Therapeutique.*

---

### The Plague in Egypt; its Causes.

From a memoir on this subject, recently read before the Academy of Medicine by M. *Hamont*, we select the following picture of the loathsome condition in which a large portion of the population of Egypt is living, even in the present day.

"I have passed," says he, "fourteen years in that country, and I have studied the disease with all possible care. It is truly endemic there; no one disputes the fact; and the cause of its being so, it is not difficult to explain. The plague reigns exclusively in what is called Lower Egypt—a region where every imaginable condition of insalubrity is found combined; and that too in a degree which it is not easy for any one to credit, if he has not himself been an eye-witness. The dwellings of the *fellah* population are constructed of mud, and are usually situated beside some stagnant and putrid water. They are common alike to men and beasts; and when night comes, all the inmates lie down together on some filthy straw, with nothing but still filthier mats to separate them. The very ordure on the floor is removed only for the purpose of hanging it up in lumps from the walls to dry: this serves for fuel! Judge of the stench that must arise from this novel species of combustible matter. Then again, the horrid miasms from putrid carcases of different animals lying round the walls, and perhaps also from the bodies of human beings buried only a few inches underneath the surface. Who, but a *fellah*, could endure such horribly offensive scenes?

"When, in consequence of the excessive mortality from the Plague, the living are insufficient to inter the dead, the bodies are carried away in heaps upon a bier to the cemeteries, and there left to rot in the open air. The villages, that are in short nothing better than a heap of privies, are usually surrounded with a belt of rubbish, as if actually to prevent all chance of ventilation! Often the traveller knows that he is approaching some dwellings, only by the very exhalations that corrupt the air around. And yet the poor ignorant inhabitants will refuse all advice or the suggestion of any change; nay, they treat as impious whosoever ventures to propose it, telling you that, do what you may, death will come at his appointed time! * * * * * *

"Three-fourths of the population live on tainted meat—the flesh perhaps of animals that have died of carbuncle—on old rotten cheese, putrid fish, leaves of radishes and other vegetables; and in some districts on rats and locusts. Such is the common food of this wretched people—the slaves of a most cruel despotism."—*Comptes Rendus.*

---

### THE THERAPEUTICS OF NATURE.

"Therapeutics, it is very generally admitted, is one of those branches of medical knowledge in which there exists the greatest amount of errors, defects, and prejudices; and where experience is alike most difficult and deceptive. The mistakes, that are daily made, are often far greater that we are willing to admit. And then, how little do we know of the extent of Nature's own curative resources, and how much she will often effect, unaided by, or perhaps even in spite of, the interference of art. In the practice of our profession, it should ever be borne in mind that we have to do, not only with the existing disease, but also with the conservative and reparatory efforts of Nature,—which, by itself, is often sufficient to produce a cure. Hence those reputations of medicines and modes of treatment, which so rapidly start up and are as quickly forgotten; and hence those false-gods of Therapeutics, that to-day are adored, and to-morrow are despised."—*Bulletin de Therapeutique.*

---

### On the occasional Pulsation of the Veins.

It is not to that pulsation of the jugular veins—which depends upon a reflux of part of the blood from the right cavities of the heart, in consequence of the imperfect closure of the tricuspid valve—that the following remarks are applicable. "Our present object," says M. *Martin Solon* in his communication to the Royal Academy, "is to draw attention to a pulsatory movement that we have occasionally observed in the veins on the back of the hand—a movement, that is dependent upon an isochronous continuation of the arterial pulse, and which, therefore, truly deserves to be considered as a pulse of the veins." The first instance, in which he observed the phenomenon, was one of severe Pneumonia, after the patient had been largely bled. On the fifth day, the dorsal veins of each hand were perceived alternately to swell up and to subside, as is the case with any superficial artery. The pulsations were distinctly isochronous with those at the wrist. It was abundantly obvious that the movements of the veins were quite independent of those of the neighbouring arteries, and of the agitation of the subjacent tendons. When the Brachial artery was firmly compressed, the pulse in the veins, as well as in the radial and ulnar arteries, was at once suspended. In this instance, the phenomenon in question was observed for six or seven days successively. M. *Solon* expressed his conviction, that the venous pulse was in short nothing else but the continuation of the ordinary arterial one, communicated from the arteries directly to the veins. It is not improbable, he very justly remarks, that, in order that this phenomenon may occur, the circulating fluids should be in a more attenuated condition than they are in a state of perfect health. That such is probably the case, is rendered still more likely by the results of the second case—one of Typhoid fever—related by him.

Dr. *Graves* also has had occasion to observe a distinct pulsation of the veins in two cases of active inflammation, in both of which blood-letting had been already freely employed; and Dr. *Ward* too has seen it in a woman recently delivered. It would seem therefore that the phenomenon in question is decidedly connected with an attenuated state of the blood.

M. *Cruveilhier* stated that he had never seen any appearance of pulse in the veins of the hand, although the phenomenon in question was not at all uncommon in the veins at the bend of the arm. He had indeed more than once, in consequence of the vein in blood-letting throwing out its contents in jets, been apprehensive that the artery was wounded; but such an accident had never occurred in his practice. He attributed the phenomenon of the venous pulse, in all cases, to subjacent arteries communicating their movement to the veins; and never to any direct transmission of their pulsations along their capillary extremities.

M. *Velpeau* alluded to a case of Typhus fever, in which all the superficial veins in both upper extremities were moved with most distinct pulsations, that were certainly not owing to any shock communicated to them directly by subjacent or neighbouring arteries. On the whole, he was inclined to attribute the phenomenon to the reflux of the blood backwards from the heart, along the subclavian and brachial veins. But, as M. *Solon* remarked, how could this explanation hold in his two cases, where there was no pulsation of the brachial and antibrachial veins, but only of those of the hands?

M. *Blandin* expressed his entire concurrence in the opinions of M. *Solon*. He alluded to the circumstance that, two centuries ago, *Harvey* himself had distinctly asserted that the heart will sometimes transmit its pulsations even to the veins: and he then mentioned some experiments which M. *Magendie* and he had performed, and which tended to establish the truth of this position. If a part of the circulatory system be emptied, (in the dead body) by means of a first injection pushed in with force, and then a second injection be thrown in, by jerks, into a large arterial trunk, we shall find that it will escape from a wound in any one of the principal veins of that system, in jets, as from an artery. If this can be done in the dead body, why should we deny the possible occurrence of it in the living?

M. *Dubois* intimated his dissent from these views, and agreed with M. *Cruveilhier* in ascribing all venous pulsations, either to the shocks communicated from the contiguous arteries, or (in some cases) to the reflux of the blood along the jugulars.—*Comptes Rendus.*

---

### ON THE USE OF THE THYMUS GLAND.

Dr. *Picci*, after glancing at the theories of his predecessors, suggests that the use of this Gland is chiefly of a mechanical nature; viz. to occupy a certain space within the thoracic cavity, while the lungs remain unexpanded in the fœtus; and thus to prevent the ribs and sternum from falling in too much upon these vital organs. The size of the Thymus is inversely as the volume of the lungs; and, when the latter become dilated after birth by the admission of air into their cells, the former immediately begins to shrink and become atrophied. In truth, it is only in the adult that the thoracic parietes are moulded completely upon the lungs; for, in infancy and youth, it is rather the Thymus gland that is, in their place, moulded upon the thorax.

The situation of this gland in the anterior mediastinum and along the median line, the very nature of its tissue, and the greater expansion and development of its inferior half, are adduced as arguments in favour of the opinion now adduced. Besides the well-known circumstance that, in those new-born children in whom the thorax is very largely developed, the Thymus continues to increase gradually even to the end of the second year, it deserves notice that all those animals, in which the lungs are similar to those in the human subject, are provided with this gland; whereas, we find it to be entirely wanting in those which breath by Branchiæ or membranous lungs. In hybernating animals, also, the Thymus exhibits alternations of enlargement and decrease, according to the state of the respiratory organs. In the Amphibia it attains its maximum of development.

The circumstance too of the gland being usually rather larger than ordinary in phthisical patients may be mentioned as lending some probability to the view we have proposed.—*Annali Universali.*

---

### SPONTANEOUS EXPULSION OF THE OS HYOIDES.

A middle-aged woman, of a rachitic constitution, became affected with an enlargement of the glands around the lower jaw, accompanied with a slight cough, and disturbance of the breathing. In spite of treatment, these symptoms went on gradually increasing, for three or four years. The sputa became thick and viscid, and were occasionally streaked with blood; the patient was every now and then distressed with attacks of suffocative dyspnœa, and her strength was greatly exhausted by colliquative sweats. The voice was at length completely extinct; and now there was a fixed pain, with an almost continual sense of pricking, in the laryngeal region. The sputa were at this time very decidedly purulent, and were often rejected without any effort of expectoration: it was a simple exspuition that followed immediately after a lacerating pain felt in the throat.

The condition of the patient had for a length of time appeared quite hopeless, when most unexpectedly—after experiencing more than usual suffering from the cough, dyspnœa and pricking pain in the throat—she expectorated, in the midst of a convulsive agitation of the whole body, a firm hard substance, which proved to be a bone of considerable size. On being examined, it proved to be the *os hyoides*. The health of the patient speedily improved, and was ultimately quite restored—five years after the commencement of her first suffering. The bone was examined by several members of the Academy, so that no reasonable doubt as to its nature can be entertained.—*Comptes Rendus.*

---

### Surgical Memoranda by M. Vidal.

I. *Luxation of the First Phalanx of the Thumb.*—The reduction of this accident is almost always extremely difficult. A variety of suggestions have accordingly been proposed to effect the object in view. Shortly after I arrived (says our author) in Paris, a case of Dislocation of the Thumb backwards was brought into the Hôtel Dieu: every imaginable attempt to reduce the displacement was ineffectually tried. M. *Dupuytren* delivered a lecture upon the accident, and endeavoured to prove that the irreducibility was caused by the altered position of the lateral ligaments of the joint: these bands, in consequence (he supposed) of having become stretched in an oblique direction, bound down the digital bone against its metacarpal fellow. Upon that occasion, I shewed that the real obstacle was a *boutonniere* of muscular substance, which strangulated the head of the metacarpal bone, and became the more and more tightened in proportion to the force with which traction was applied to the displaced phalanx. This impediment is formed on the outer side by the external portion, and on the inner side by the internal portion, of the small *flexor pollicis* and *adductor brevis.* As the heads of these muscles are inserted into the upper extremity of the first phalanx, they are necessarily forced backwards along with the dislocated bone, and the upper metacarpal protuberance is thus held fast between them. To effect the reduction, tractions in a variety of directions had been (as already mentioned) tried for a length of time; but all in vain. I proposed to cut away the head of the metacarpal bone; but the suggestion was not, and perhaps wisely, adopted. M. *Malgaigne* has more judiciously suggested that it would be better to divide the external portion of the *boutonniere* or strangulating muscular band.

II. *Vesico-Vaginal Fistula.*—M. *Vidal* has proposed, in unmanageable cases of this most distressing accident, to obliterate the orifice of the vagina entirely, so as to convert this canal into a blind passage, and thereby to prevent the issue of the urine from it. This idea was suggested to him by the accidental effects produced by the nitrate of silver having been applied rather too freely to the anterior and posterior walls of the vagina, in a case where the use of a suture to the fistula had failed, and the application of the caustic had been tried instead: the opposite walls of the vagina joined together, and quite prevented the escape of any fluid from its orifice. The result was that the urine flowed from the urethra. This state of things continued for nearly a fortnight. Unfortunately, upon endeavouring to ascertain, by means of a manual examination, the state of the parts, the feeble adhesion of the vagina gave way, and the urine again began to escape from its orifice.

In this, and indeed in almost every case of vesico-vaginal fistula, a great obstacle to the cicatrization of its edges is the fact that the bladder has usually become so contracted upon itself that it is unable to hold any considerable quantity of urine. The fluid is therefore continually escaping from the ulcerated opening; and, when the discharge is obstructed by suture, the patient is distressed with most frequent calls to micturition. The effects thus induced very materially interfere, as a matter of course, with the process of healing: and unfortunately too, the leaving of a catheter in the bladder does not answer well, in consequence of the contracted state of the cavity of this organ.

M. *Vidal* has once carried his proposal into effect, in the case of a woman, 35 years of age, in whom the vesical fistula was so large as to admit the introduction of several fingers through the ulcerated opening, into the bladder. After paring the edges of the vulvar orifice of the vagina, he passed three stitches through the internal *labia*, and tied them over two pieces of bougie, in the usual manner of forming a quilled suture. Next day, the urine was voided by the urethra; and, for nearly a month, not a drop made its escape from the vagina. The catamenia also, during this period, passed along with the urine from the urethra. Most unfortunately, a difficulty in passing the water having come on, one of his pupils made an attempt to introduce a catheter into this canal and pressed too strongly against the vaginal cicatrix. The result was that the adhesion gave way; some blood was discharged at first, and subsequently urine came away by the wound.

M. *Vidal* closes his remarks in these words :—"The difficulty of closing the orifice of the vagina is indeed real; but to say that it is impossible is nothing but a vague assertion. The objection that I deprive the woman of 'son plus bel attribut,' that I rob her of her sex, and prevent her from ever conceiving afterwards, is one that it is quite needless for me to reply to."

### III. *Lithotomy 'en plusieurs temps.'*

In my opinion, says our author, the infiltration of the urine into the surrounding tissues is the most formidable accident that is liable to occur after this operation. After a wound of the bladder, the urine comes immediately in contact with a loose, and therefore very infiltrable, cellular tissue. Now, if nature does not re-act with energy, and if there be not promptly formed a sort of protecting eschar upon the surface of the incised parts, the effusion of the urine will take place, and death is then almost inevitable. To prevent this accident, it should be our effort to render this cellular tissue less lax and permeable; and this can only be done, with any prospect of success, before the urine is allowed to escape from the bladder. *Eh bien!* this most desirable result may be obtained, if the operation be performed at several times; if the skin and subjacent tissues down to the bladder be divided at first; and the section of the bladder be not made until after the organic re-action, which changes the physical and vital conditions of the surrounding parts. The first time or step in the operation, in some cases and in certain subjects, might be effected by means of caustic; but certainly the knife is very generally to be preferred.

### IV. *'Debridement Multiple' applied to the Operation of Strangulated Hernia.*

M. *Vidal*, after having pointed out the advantages of the bilateral and even the quadrilateral incision of the Prostate Gland in some cases of Lithotomy—proceeds to point out the applicability of a similar method of proceeding in the operation for relieving a strangulated bowel. Every one is aware that, in certain cases, where there is an anomalous distribution of the *epigastric and obturator* arteries, great danger of an alarming hæmorrhage is incurred, if the incision of the ring or aperture be prolonged too far in any one direction. Now this risk may, in a very great measure, be avoided by making a number of nicks or little incisions, in place of a single one, that is more free and extended. It is unnecessary to enter into further details; all that is necessary is to suggest the hint.

(The hint is far from being a novel one; it will be found in most of the standard works on Operative Surgery).

### Remarks on Ambulant or Erratic Erysipelas.

"The epithet applied to this form of Erysipelatous inflammation designates its characteristic feature. The cutaneous affection propagates itself by irregular impulsions. In one case it attacks a part which it speedily quits, without passing through its successive stages; and in another, the disease exhibits all the phases of its complete evolution in the part that is primarily seized. Not obedient to any regular march, it leaps from one region to another, without implicating the intermediate parts; or it suddenly vanishes from the skin, to fasten itself upon some internal organ. The inflammatory action usually does not penetrate deep; it but skims the cutaneous surface. The danger, therefore, of this form of Erysipelas arises not so much from the extent or severity of the dermal lesions, as from the risk of metastasis of the morbid action to some important viscus, and from the cachectic state of the general health.

Erratic Erysipelas may terminate in various ways. In the majority of cases, it proves fatal. It very rarely terminates by simple resolution and by a *direct* return to health. A fortunate termination of the disease is usually accompanied or followed by some critical evacuation, as a hæmorrhage from the nose, a profuse perspiration, a copious flow of dark-coloured urine, or by a severe diarrhœa.

Occasionally—but this termination has been but little attended to—the critical effort seems to consist in the development of numerous subcutaneous Abscesses, unpreceded by any inflammatory action in the parts so affected.

This lesion must not be confounded with the suppuration of the subcutaneous cellular tissue, that is not unfrequently the consequence of *phlegmonous* Erysipelas: in the *ambulant* form of the disease, the inflammation is strictly superficial, and the subjacent cellular substance is not involved in the morbid process.

In the two cases related by *M. Tanquerel,* erratic Erysipelas had continued for upwards of three weeks, and, being accompanied with Typhoid symptoms, threatened great danger; when, suddenly, a number of small indurated tubercles were perceptible under the skin of the neck and chest—the cutaneous inflammation having left these parts a few days before, and attacked the lower extremities. On the following day, many of these tumours presented a distinct sense of fluctuation. In one case, as many as forty-three of these boils or subcutaneous abscesses formed in the space of three weeks. The size of them varied from that of a small nut to that of a pigeon's egg.

They were developed without any previous heat, pain, or redness in the parts affected; and even their existence was often not recognised, before it was found that purulent matter was already formed in them.

From the moment that the earliest abscess was formed, the Erysipelas ceased to make any further advance beyond the part where it happened then to be; and soon afterwards it ceased altogether.

We need scarcely say that the convalescence from such an attack must always be very tedious. There was no reason to suppose that, in either of the cases which occurred to Dr. T., there was any existing Phlebitis,—a lesion which is so often associated with, if it be not the immediate cause of, those *metastatic abscesses* which are generally referred to what has been called, of late years, a Purulent Infection of the System. It is more than probable that there is always present, in both sets of cases, a morbid alteration of the circulating fluids; although we are not yet prepared to say in what this alteration consists."—*Journal de Medecine de M. Beau, Sept.* 1844.

### PROFESSOR TOMMASINI ON CHRONIC ARTERITIS AS A CAUSE OF MANY DISEASES.

The intimate connection between Rheumatism and an inflamed condition of the heart and arterial blood-vessels has been recognised by many of the most eminent pathologists of the present day. *Rasori*, indeed, has denied that the arteries are susceptible of inflammation; but in this opinion he is unquestionably wrong.

"Since the year 1806," (we quote the words of the proto-physician of Parma,) "my attention has been directed to those morbid vibrations which the arteries sometimes exhibit, and which often occur without being accompanied by any heat or dryness of the surface, redness of the face, or any other symptom of fever. This phenomenon is announced by a monotonous frequency of the pulse, continuing for months, and, it may be, for years; the patient perhaps all this while presenting no symptom of organic disease in any part of the body; and even dissection (for I have seen a good many die in this condition) not disclosing any satisfactory alterations of structure, to account for it."

These arterial vibrations are of frequent occurrence in Chlorotic females, in whom the catamenia have been for some time irregular, and in patients who have lost much blood, either by spontaneous hæmorrhage or by artificial depletion. They are occasionally observed also in the large arteries of an amputated stump, after all the fever and suppurative inflammation have ceased. In women too who have been recently confined, and in children subject to Epistaxis, the pulse is generally found to be more or less vibratory and rapid: the same occurs after frights and great mental disquietudes, in cases of Dropsy of the Pericardium also, and in Hysterical and Hypochondrical affections. In the last-named maladies, the patients are often affected with such violent vibratory pulsations in the Epigastric region, that they fancy that they must be labouring under aneurism of the abdominal aorta.

"The question now comes to be," says the Professor, "what is the cause of the phenomena alluded to? In no work of medicine, ancient or modern, is there, to my knowledge, any satisfactory explanation of it proposed.

"In my opinion, it is attributable to a chronic phlogistic condition of the vascular system: the experience and observation of many years have tended to confirm more and more my belief in this doctrine. The inflammatory action supposed to be present, is—be it remembered—not only chronic in its nature, but in the *minimum* degree of force: it may therefore continue for a great length of time, without inducing almost any disturbance of the general health. But if it be aggravated by injudicious treatment, organic changes of the affected tissue may be gradually brought on."

"I have remarked," continues he, "in all diseases termed leucophlegmatic by the older physicians, and especially in Chlorosis, in which there is so marked a tendency to œdematous tumefaction of the cellular tissue, that there is a slow chronic arteritis, which in my opinion is to be regarded as the cause of all the phenomena of the malady. The vibratory character and great frequency of the pulse are symptoms well known to every medical practitioner. There is the most intimate alliance between the leucophlegmatic colour of the skin and the swelling of the cellular tissue, and this phlogistic condition of the arterial system; although, on first consideration, we might anticipate a very different result, and expect that, as the circulation of the blood is so very rapid, the lymph, instead of being effused into the cellular substance, would be carried along in the sanguiferous vessels."

Several cases are relatad with the view of confirming these pathological views. We shall give brief abstracts of two of them, before we offer any criticisms.

*Case.*—A man, who had been long addicted to the use of stimulating liquors, was seized with dizziness and other symptoms that indicated plethora in the head. He was freely bled, and kept on a low diet. A month or two afterwards, his appearance was very seriously altered; he was then pale and had a chlorotic aspect; the skin was puffy and seemed to be bloated; he complained of a distressing sense of heat in the chest; his pulse, which was formerly soft and slow, was now exceedingly rapid and had a sort of *metallic vibratory* movement; but there were no palpitations of the heart present, nor any decided difficulty of breathing; except indeed upon any bodily exertion or mental disquietude; and then the heart throbbed violently, the breathing was hurried and distressed, and the vibratory character of the pulse was unusually strong. I advised him to be bled, to take pills of aloes and squills, and a chalybeated cream of tartar: the patient, however refused to be bled, as he attributed all his present suffering to the large losses of blood which had been practised on a former occasion. Subsequently he died of Hydrothorax. "I feel convinced (says *Tommasini*) that, if the body had been examined, the chief morbid appearances would have been an inflammatory state of the arterial system, indicated by redness and congestion of the inner surface of the blood-vessels, thickening of their parietes, and transudations here and there of concrescible lymph."

*Case* 2.—A young nobleman had for some time been subject to attacks of violent hemoptysis, but without ever exhibiting any of the characteristic symptoms of Phthisis. He had no cough (except when these attacks occurred), nor fever; and yet his pulse was more vibratory, metallic and thrilling than I had ever felt before: it was also very rapid. Blood, drawn from the arm, was so highly fibrinous that it became covered with a thick buffy coat, in an unusually short space of time. Besides these well-marked symptoms of Arteritis, this patient exhibited all the appearance of a genuine Chlorosis. He died during a severe access of pulmonary hæmorrhage.

The Professor admits that most medical men would have attributed the vibratory character of the pulse and the other symptoms in the preceding cases to the weakness and atonic irritability of the vascular system, induced by the losses of blood; but he argues against this opinion on the following general grounds. How comes it for example, that a young girl, previously in good health, should, in consequence of the stoppage of the catamenial discharge, become affected with Chlorosis? Surely the mere arrest of this sanguineous evacuation ought not to induce the characteristic features of this disease. Again, do we not observe that a patient, affected with any acute inflammation, may lose ten times the quantity of blood—in the same, or even a shorter, space of time—that another patient loses spontaneously, *i. e.* by some hæmorrhagic disease? and yet the former is not found to exhibit the chlorotic appearance, and the vibratory condition of the circulation which are so often observed to exist in the latter. "I do not mean to deny," says *Tommasini*, "that these phenomena may be induced by excessive sanguineous depletions; but what I insist upon is, that often, in hæmorrhagic patients, we confound the effects of the loss of blood with the pre-existing and essential condition of the malady; and that, in very many cases, the leuco-phlegmatic habit is intimately connected with a morbid condition of the blood-vessels, in short with a chronic inflammatory state of these parts."

The altered state of the blood is attributed by our author, in part, to the morbid state of the vessels themselves, and in part also to the disturbance of the digestive function; and the tendency to the serous effusions is made to depend upon the intimate alliance between the blood-vessels and the lymphatics, so that whenever one system becomes disordered, the other invariably suffers.

Professor *Tommasini* then adduces a multitude of cases of Hypochondriasis and Hysteria, in which—alike from the symptoms during life, and (in certain cases) from the morbid appearances found in the aorta on dissection—he contends that there was a chronic inflammation of the arteries present.

"For the last thirty years," says he, "I have taught in my lectures that chronic Angeioitis is the principal cause of flatulences, nervous disturbances, and hypochondriacal affections, or, at least, that it is most intimately connected with these morbid states; and certain it is that I have often diagnosticated the presence of the lesion in question in cases, where it was never suspected by the other medical attendants, and have afterwards positively ascertained its existence by dissection, to the surprise of those who followed my clinical practice at the time. In most of these cases, the abdominal aorta pulsated with a strong metallic vibration, the complexion of the patient was unhealthy and pale, and the digestive functions were more or less disordered. * * * * * In spite of the symptoms of gastritis, dyspepsia, flatulence, gastralgia, hypochondriasis and hysteria that were present, I have often detected the cause and source of all the existent mischief, by merely laying my hand upon the epigastric region."

He subsequently qualifies these statements very materially, by admitting that all the phenomena of these diseases may be present, without any real or idiopathic affection of the arterial system; but nevertheless he re-asserts with earnestness his opinion that Angeioitis is of much more frequent occurrence than is generally admitted by pathologists.

The numerous and intimate connections between the nerves that supply the heart and the large intestines on the one hand, and the abdominal viscera on the other, are sufficient, he thinks, to account for the close sympathies that exist between the functions of the Circulatory and Digestive organs, in various diseases, as well as in a state of health.—*Annales de Therapeutique et de Toxicologie.*

*Remarks.*—The pathological doctrines here propounded are, on the whole, so utterly at variance with those almost universally held in this country, in respect to the diseases alluded to, that it is scarcely necessary to do more than simply direct our readers' attention to the subject. That Chlorosis and hæmorrhagic diseases, when accompanied with a vibratory pulsation of the heart and great arteries, and a tendency to subcutaneous œdema, are generally to be regarded as the effects of a slow chronic Angeioitis, is a position that few English physicians will admit. The practice of Professor *Tommasini* is a little better than his theory. Occasional bleedings, and the use of digitalis, squills, colchicum, nitrate and acetate of potash, aloes, and steel (for this also is considered to be a *contra-stimulant*), are the chief remedies which he recommends. With respect to this alleged action of steel, it may be worthy of notice that we have frequently had occasion to remark, that the opinions of Italian physicians, as to the action or *modus operandi* of certain medicines on the system, are quite antipodal to those held by the medical men of other countries. In one of the Numbers of the *Gazetta Medica di Milano,* during the last year, we find a Dr. Mattii publishing several cases of Pneumonia and other actively inflammatory diseases, in which he exhibited the *secale cornutum* with (he says) decided advantage: its action, according to him, being *hyposthenic,* and not stimulant, as is generally imagined.—(*Rev.*)

